# The Dynamic Relationship between Social Cohesion and Urban Green Space in Diverse Communities: Opportunities and Challenges to Public Health

**DOI:** 10.3390/ijerph21060800

**Published:** 2024-06-19

**Authors:** Viniece Jennings, Alessandro Rigolon, Jasmine Thompson, Athena Murray, Ariel Henderson, Richard Schulterbrandt Gragg

**Affiliations:** 1 School of the Environment, Florida Agricultural and Mechanical University, Tallahassee, FL 32307, USA; richard.gragg@famu.edu; 2 Department City and Metropolitan Planning, The University of Utah, Salt Lake City, UT 84112, USA; 3 Department of Public Health, Agnes Scott College, Decatur, GA 30030, USA

**Keywords:** urban green space, public health, social cohesion, nature, diverse communities

## Abstract

Social cohesion is a key factor within social determinants of health and well-being. Urban green spaces can provide environments that potentially facilitate meaningful and positive social interactions that promote social cohesion, equity, human health, and well-being. However, the lack of integration of existing research on social cohesion, urban green spaces, and public health in diverse (e.g., racially and ethnically) communities limits culturally relevant strategies to address health disparities. In this manuscript, we synthesize research on the potential of urban green spaces to promote social cohesion and public health in historically excluded communities. Particularly, we explore the development of social cohesion as it relates to the social environment, built environment, leisure opportunities, green space maintenance, safety, and green gentrification. We highlight key factors and their application to meet opportunities and challenges to social and public health. A conceptual framework is presented to provide an overview and illustrate connections found in the literature.

## 1. Introduction

A key pathway through which urban green space (e.g., parks, gardens, and forests) can promote health benefits is the potential development of social cohesion while people visit parks and other open spaces [1]. Social cohesion can be described as the shared norms, values, and interpersonal dynamics that can indicate quality of life and feelings of belonging [1,2,3,4,5]. The provision of urban green spaces is linked to social determinants of health and ecosystem services that can support human health and well-being [1,6,7,8,9,10,11]. Jennings and Bamkole [12] developed a framework to conceptualize the relationship between ecosystem services from urban green spaces, social cohesion, and public health. Specifically, urban green spaces can promote social cohesion through place attachment, social support, belonging, and empowerment, as well as physiological and behavioral responses that promote health [12,13]. Previous research acknowledges that the underlying social environment characterizes opportunities for people to develop (or hinder) social cohesion [12,14,15,16] and health promotion in urban green spaces [1,9,12,17]. Factors that can enhance social cohesion include improving the maintenance of urban green spaces, reducing crime, the presence of amenities that support social gatherings, perceived safety, accessibility, and cultural activities [18,19].

While urban green spaces can promote social cohesion and public health [1,12], stressors can worsen quality of life and contribute to health disparities [20]. As social connections for many people were often limited and worsened during the COVID-19 pandemic, it is important to strategically cultivate knowledge across disciplines to address gaps in evidence in the United States and globally [21]. The interaction between the social environment and green spaces can impact diverse populations around the world. For example, studies have explored the relationship between parks and social interactions in the United States [22,23], India [24,25], China [26,27], the United Kingdom [28], South Africa [29,30], Colombia [31], Italy [32,33], Ghana [34], and Australia [35]. These studies can also inform how the social environment relates to experiences that racially/ethnically diverse populations have in green spaces. Conversely, members of diverse communities often experience cultural, discriminatory, and economic barriers that affect their interaction with nature [28] and other people.

Furthermore, loneliness, isolation, and the lack of social connectedness are major public health issues that are often underrecognized [21,36]. A recent U.S. Surgeon General’s report describes the importance of social cohesion in the healing effects of social connection [36]. Researchers also describe how the availability of quality urban green spaces can increase social interactions and potentially buffer the effects of loneliness [37,38,39]. As the relationship between urban green spaces and loneliness can be mediated by social cohesion [40], understanding the nuances of this interaction may be particularly important in diverse populations [19,41] that experience forms of social exclusion. Thus, unique factors may explain how green space is associated with social cohesion among diverse communities.

As scholars identify the importance and call for research on urban green spaces and social cohesion in diverse communities [12,18], this manuscript bridges this knowledge gap to advance our understanding of the topic. Given the presence of diverse populations and demographic shifts taking place in countries such as the United States [42], exploring the experiences of historically excluded populations on urban green spaces is salient for improved research and practice [43,44]. Within the context of this article, we define historically excluded populations as racially/ethnically diverse communities that have and are disenfranchised by social injustice. In this synthesis article, we explore existing research on social cohesion, urban green spaces, and public health, particularly in diverse communities. As previous research on green spaces and social cohesion identified leisure, green gentrification, safety, maintenance, and other mediators as areas for additional research [12,18], we synthesize studies in diverse communities related to social cohesion and urban green spaces. We then present a discussion of key findings and a conceptual framework followed by areas for future research. This insight strategically bridges transdisciplinary findings to elevate such knowledge for research, practitioners, decision makers and diverse communities at the intersection of these topics.

## 2. Literature Areas Elucidating the Relationship between Green Space, Social Cohesion and Health for Diverse Communities

In the following sections, we discuss six literature areas that inform the relationships between green space and social cohesion among diverse communities. We examine some of the related connections and highlight how physical and social characteristics related to urban green spaces encourage or hinder social cohesion.

### 2.1. The Social Environment and Public Health

Social cohesion can greatly characterize conditions within the social environment [3] which relate to public health and well-being. From a theoretical perspective, research on neighborhood resource-based theory describes how social cohesion relates to conditions, behaviors and stressors that affect human health [45]. Extensions of the theory of human motivation [46,47] and network theory of social capital [48] also inform this topic. Scholars describe how the social environment relates to biological pathways linked to public health [49,50,51,52,53,54]. For example, a strained social environment can relate to health concerns such as depression [55,56], cardiovascular conditions [57] and obesity [58,59]. Studies among diverse populations have explored social cohesion on mental health among Asian Americans and Latinos [60], physical activity [61], cardiovascular conditions [62], adolescent health [63], smoking among African American women [64], and sleep disparities [65,66]. For example, low levels of neighborhood social cohesion were linked with less sleep in a population of Native Hawaiians and Pacific Islanders [65]. During a study in Maricopa County, Arizona, USA, scholars explored the link between social cohesion, neighborhood contexts and health outcomes for Hispanic and non-Hispanic residents [67]. They found that neighborhood social cohesion provided a protective factor for residential health (e.g., mental health) and accounts for some health differences associated with socioeconomic status and ethnic diversity [67].

Along with the health implications, the social environment relates to the interactions and cohesiveness experienced by diverse populations. Mulvaney-Day et al. [68] analyzed the interconnection between social cohesion, social support, and health among Latinos in the United States. Using data from the National Latino and Asian American Study (NLAAS), they found that economic status and education level can also relate to both social connections and health within the Latino population [68]. Other cultural factors were involved in this interaction. For example, language capabilities were strongly tied to social connections related to physical health among Latinos [68]. They concluded that sociodemographic status plays a role in neighborhood social cohesion and access to resources that align with health promotion [68]. Others observed cases where social cohesion was rated lower among individuals with foreign citizenship [69]. Similarly, the length of residency and higher acculturation were linked to more visits with friends by Hispanic immigrants to urban forests in Southern California [70]. However, opposite observations amongst Puerto Rican communities in Massachusetts [71] and Hispanic Americans in California [72] showed that observations can vary. Hong et al. also observed that residents with a favorable perception of neighborhood social cohesion were more likely to have better mental health [73]. However, they observed variations by ethnic group. For example, Asian Americans in areas with greater intragroup density had a lower perception of neighborhood social cohesion while some Latino neighborhoods with a high density of other Latinos were perceived to be more socially cohesive [73].

Urban green spaces combined with a favorable social environment can support community engagement, social capital and community satisfaction in ways that promote health equity [74]. However, in the same way that social cohesion develops from a multi directional fashion [12], the factors that solidify and diminish social cohesion in diverse communities are important. During a study on urban green spaces and health in Europe, diverse respondents (e.g., African-Caribbean or Bangladeshi origin) had lower levels of physical activity and perceptions of worse health which may relate to a lower sense of belonging, trust and perception of local areas [75]. The consequences of institutionalized and structural racism relate to inequitable access to urban green spaces [76,77] as well as the manifestation of power and privilege in many parks systems [76]. Historical and contemporary social challenges can strain the interaction that racially/ethnically diverse communities have in nature [76,78]. Scholars describe the elitism and racism of some major conservation groups resulted in “public parks by displacing, excluding, and criminalizing the Indigenous, the poor, people of color, and immigrants” [79]. For instance, a study in Los Angeles, California found that some Latinos experienced accessibility barriers to park use, including distance, not feeling welcome and anxiety to visit white neighborhoods [80]. Ultimately, the author concluded that less exposure to green spaces (e.g., access or quality) is associated with racial residential segregation [80].

### 2.2. Characteristics of the Built Environment

The presence, design, and quality of the built environment play a major role in health promotion [81,82]. Previous studies describe how the built environment relates to the development of social cohesion [69], social capital [83] and determinants of health [9]. Studies have also linked food access [84], walkability [18,83] and transportation [18] to a quality built environment and subsequent health-promoting behaviors. For example, a study found that Latinos who reside in areas with higher levels of social cohesion were significantly more involved in physical activity [85]. However, others observed that social cohesion can decline due to urbanization [69], which can relate to conditions within the built environment. For example, short distances to the city center, local amenities, and high neighborhood density had a negative association with social cohesion yet transportation was positively associated with social cohesion [69].

The social experiences of racially/ethnically diverse populations can be linked to the design, management, and exploration of their physical landscapes [86]. A study on urban parks and social cohesion articulates how location, park design, personal perception and cultural preferences contribute to social interactions [39]. For example, research describes how neighborhood walkability and high-quality parks are linked to social capital and park satisfaction in diverse communities [83]. While the availability of urban green spaces is important, a study in Singapore observed that a sense of belonging and inclusive community reinforced the positive relationship between gardens and social cohesion [87]. Other research with racially/ethnically diverse study participants describes how the availability of parks relates to greater social capital and park use [88]. Similarly, research on home gardens in San Jose, California discussed how their presence provided food access/security, increased vegetable consumption, and an increased perception of being a part of the community for Latin American residents [89]. The availability of community gardens can positively influence social connections and increase participation in activities that enhance physical health among vulnerable populations such as refugees [90].

However, inequities within the built environment can hinder the use of parks and access to their social benefits [83]. For instance, inaccessible amenities in the built environment can contribute to social isolation in older adults [91]. Through the years, scholars describe how residential segregation relates to inequitable access to a quality built environment, exposure to pollution [92,93,94], access to urban green spaces [95,96], blighted vacant land [97], and various health concerns [98,99].

The residue and contemporary tactics of segregated spaces relate to an impaired social environment and interpersonal conflicts that can occur in urban green spaces. Studies describe how residential segregation relates to an inequitable distribution of canopy cover [96], parks, and overall green spaces [100,101], resulting in environmental injustice concerns [95,102,103,104]. Such inequitable access to urban green spaces can linked be to health disparities in heat-related illness [96], psychological wellness, obesity and cardiovascular health [105]. A literature review explored the association between racial residential segregation and greenness exposure in the United States [80]. The analysis found that areas with more Black residents had significantly lower amounts of tree coverage while areas with a large Hispanic or Asian population had no tree coverage [80]. Inequalities in the built environment can hinder leisure options for residents in many underserved communities. While increased physical activity was encouraged during the early years of the COVID-19 pandemic, people who already resided in walkable communities were more likely to engage in walking activities [106]. A study across some metropolitan areas in the western U.S. showed that low-income residents from diverse communities had worse park access compared to more privileged groups [107]. Furthermore, another study showed that insufficient amenities for social gatherings and previous instances of discrimination related to feelings of park exclusion among Muslim women in the United Kingdom [28].

Access to quality green spaces coupled with a quality social environment promote opportunities to engage in health-promoting behaviors (e.g., physical activity) that relate to social cohesion [85]. Amenities within the built environment can also be linked to social cohesion and interaction with green spaces. A study in China observed that a short walking distance, open landscape and wheelchair accessibility to gardeners were linked to higher social capital while longtime gardeners displayed stable social connections [108]. Similarly, people who reside in walkable neighborhoods are more likely to be engaged in their community and know their neighbors [109]. Although urban green spaces can promote walkability [110] and related social interactions [12], the characteristics of the built environment (e.g., neighborhood perception, walkability, traffic, safety from crime) can affect how park benefits are relayed across diverse populations [83]. For instance, streets with bike infrastructure can reduce the likelihood of cyclists receiving a ticket and possibly having a negative experience with police [111]. Areas with a larger population of people from diverse backgrounds had more walkability to parks [80].

### 2.3. Leisure in Urban Green Spaces

Involvement in outdoor recreation and leisure satisfaction can be positively associated with social well-being [112]. Social cohesion can contribute to engagement in leisure activities that support physical activity and psychological well-being [113] and vice versa. As reports describe an increase in racially/ethnically diverse communities visiting parks and engaging in outdoor recreation [114], this documents the value of such experiences to these communities. A study of diverse neighborhoods in Barcelona showed that participation in leisure-based community activities was positively related with perceived neighborhood social cohesion [115]. Findings articulate that social cohesion was achieved among the neighborhoods through the collective recognition of their diverse identity [115]. As social well-being can be positively associated with identity expression [112], comfortability with urban green spaces can cultivate positive experiences with people from diverse backgrounds. As inclusion efforts, shared values with other users and places to gather can encourage social cohesion [18], these factors also relate to nature-based leisure in diverse communities. This aligns with observations that social well-being had a significant positive association with leisure satisfaction and identity affirmation [112]. Thus, personal identity can relate to one’s cultural expression as well as social and leisure preferences.

The availability of quality green spaces and the underlying social environment can be linked to leisure activities. For example, city parks can promote community well-being through a sense of local pride, engagement and safety [8]. Wan et al. [13] conducted a systematic review to explore factors involved in the relationship between public urban green spaces and social cohesion. The authors observed that inter-racial friendships tended to develop when green spaces were perceived as close [13]. Also, older people with a favorable perception of green spaces tend to have more social contact with neighbors and greater participation in outdoor activities [13]. Frequent visits to parks and participation in park organizations can also strengthen the perception of social cohesion [116]. Moreover, people who were more familiar with green spaces were likely to interact with people of different ethnic groups [13].

Other studies have explored leisure in diverse communities [112,117,118]. In North Carolina, the effect of outdoor recreation on the social well-being and leisure satisfaction amongst African Americans was explored [112]. They found that enjoyment, pleasure, and attraction from outdoor activities determined leisure satisfaction and social well-being in African Americans [112]. Similarly, social cohesion was positively associated with park ownership among African Americans in Philadelphia at a significant level [119]. Martin et al. [118] also describe the importance of representation in the outdoors, addressing safety constraints (e.g., amongst African American women), and the perception of equity within parks. Others also documented how park design or renovations can improve social interactions that lead to a park becoming a shared asset for a community [119]. Similarly, amenities, social factors and accessibility routes can influence the opportunities of children from diverse cultures to engage in outdoor play [120].

Other research has analyzed the role of urban green spaces to strengthen social cohesion in Latino and immigrant populations [121]. Murillo et al. [85] examined if engaging in leisure walking and seeing others walk influenced neighborhood social cohesion across the United States. Particularly, they explored this relationship between leisure walking and social cohesion among Latino adults [85]. They observed that fifty percent of Latino adults who engaged in leisure walking and developed neighborhood social cohesion regularly saw people walk every day [85]. Ultimately, the authors found that watching others engage in leisure activity could contribute to participation in such activities as well [85]. Abramovic et al. [122] researched the role of community gardens in the recovery of diverse refugee communities in Canberra, Australia. They observed that the garden’s ability to provide a space for refugees to feel safe, apply their own knowledge and skills to new land (in an adaptable method), share knowledge and produce food were beneficial [122]. Overall, they found three categories of benefits that gardens provide to refugee communities: the garden as a “safe space”, the garden utilized as a place of experimentation, and a place of attunement [122].

While the studies in this section discuss favorable experiences, we acknowledge the challenging nature-based leisure experiences by people from diverse communities. Dietsch et al. shared perspectives of violent oppression and racialized trauma experienced by diverse participants in nature-based leisure [123]. Negative social experiences in nature-based leisure can limit the opportunity for diverse communities to experience the breadth of social cohesion benefits tied to urban green spaces. A study shared that racially/ethnically diverse participants had traumatic experiences related to unequal treatment by authorities, environmental racism, unwelcoming behavior and cultural stigmas that strained nature-based leisure [123]. Others describe how anti-Asian racism during the COVID-19 pandemic [106], discrimination toward Mexican Americans [124], and the inappropriate policing of African Americans [125] can discourage leisure use of urban green spaces. Minimizing the impact of traumatic experiences in nature-based leisure can perpetuate biases and cultural imprints that must change for all people to have fair and equitable leisure opportunities [123] and opportunities for health promotion.

### 2.4. Maintenance of Urban Green Spaces

Maintenance can affect the likelihood of people to visit parks and engage in activities [124]. Parks that are well-maintained are more likely to bolster social benefits [18,83]. During a scoping review on the link between urban green spaces and social health, Huang and Lin [126] analyzed the findings of sixty studies on the topic. The social health benefits of green spaces can be linked to the perception of factors such as facilities, maintenance, security, aesthetics, accessibility, attractiveness, and duration of visits [126].

While residents can have multiple perceptions of green spaces, a study found that a common theme of various groups was to avoid neighborhood neglect [127]. Physical aspects of the environment (broken equipment, trash, lack of maintenance, improper lighting, traffic and busy roads) also discouraged or prevented people from going to parks [128]. For example, others have explored the link between green space maintenance to the perception and presence of crime [129,130]. Green spaces that are well-maintained are often perceived to be safer [131] and considered ‘clean spaces’ that can cultivate order and peace [127]. However, previous research describes disparities in funding for urban parks and recreational facilities in the Los Angeles area [132]. Disparities in park funding may also relate to the maintenance and aesthetic appeal of green spaces in diverse communities [124]. For example, a comparative study near Birmingham, Alabama described disparities in park features, including in care and maintenance, between two areas that varied by race/ethnicity and income [133]. Due to the ability to collect taxes that can be spent on parks, they shared that affluent communities can allocate more resources to maintain parks and manage unexpected hazards [133]. Similarly, comparative studies in Greensboro, North Carolina [134] and other locations found that park features in non-Hispanic White areas were cleaner than racially/ethnically diverse areas [135].

### 2.5. Safety on Urban Green Spaces

Safety is a major facilitator of social cohesion and the lack of safety is a key constraint [128,136]. Previous studies characterize safety through factors such as pedestrian safety, traffic, presence of streetlights, robbery, and design of the built environment [137]. Residents with favorable perceptions of neighborhood safety and social cohesion often experience significantly lower levels of perceived stress [138]. Conversely, crime, vandalism and other forms of neighborhood disorder can hinder social cohesion [18]. For example, the fear of crime and lack of safety also results in behavioral changes that negatively impact health. Residents with a fear of crime often exercise less, visit fewer friends or participate in fewer social activities [139]. However, informal social connections can buffer the negative effects of perceived neighborhood disorder and distrust [140]. As reducing social disorder (e.g., adults fighting or arguing in a hostile way) can be a neighborhood intervention to increase physical activity, it may minimize the risk of cardiovascular disease and obesity [141].

Detangling the role of multiple safety variables is important in research and practice. For example, the presence of green spaces (e.g., street trees and parks) was negatively related to social capital for neighborhoods that were perceived to be less safe [137]. As some research did not find social cohesion to be protective to health in unsafe areas [136], this suggests the importance of addressing crime to support positive community relations and health. For example, perceived neighborhood safety was linked to greater park use and physical activity amongst African American youth in Newark, NJ, USA [142]. Another study on urban park safety among low-income and racially diverse residents identified two key factors that support a sense of personal safety: social interactions and the structural environment [128]. These factors include features within green spaces (e.g., lighting, safe parking, trail improvements) and perceived social cohesion (e.g., friendliness, quality of social interactions) [128].

Safety also plays a key role in the dynamic between urban green spaces, health benefits [137,143,144], and the development of social benefits [83]. While fear of crime can also be a barrier to green space use, crimes that target racially/ethnically diverse individuals (e.g., hate crimes, harassment, and discrimination) are important considerations in social cohesion. Unfortunately, the multifunctional use of urban parks also positions them to be spaces where diverse residents may experience social injustices. These injustices can also relate to how police enforce racial hierarchies and power imbalances imposed upon people of color [86]. Racially and ethnically diverse residents are often more policed and under surveillance in parks which often leads to citations, arrests and in some cases, even death [86]. For instance, research on Chicago’s Bloomingdale Trail observed how community policing efforts targeted diverse youth and led to more non-emergency calls to the police [145]. Similarly, discrimination and inter-racial conflict upon Mexican Americans [124] along with disproportionate policing (i.e., biking related tickets) in census tracts with a majority of Black and Latino residents [111] were major barriers to park use in Chicago [124]. Moreover, some Black residents also reported that the presence of crime coupled by poor interactions with the police hindered park use and the perception of parks being a health-promoting space [125].

Hoover and Lim [86] examined privilege and power in U.S. parks during racial tensions that overlapped the COVID-19 pandemic. While neighborhood cohesion can buffer perceived discrimination, a study found that anti-Asian racism led to more discrimination and less walking activity amongst Asians during the COVID-19 lockdown [106]. The authors concluded that perceived discrimination negatively impacted walking behaviors during the pandemic and neighborhood social cohesion [106]. Other studies also observed inequalities in park use by racially/ethnically diverse communities during the COVID-19 pandemic [146]. These observations expand the current dimensions of the link between safety, urban green spaces and social cohesion in racially/ethnically diverse communities.

### 2.6. Green Gentrification

Although green space can increase social cohesion among diverse communities, new green space projects may prompt gentrification and possibly displace renters, which strains social cohesion [74,147]. For instance, recreational activities and aesthetics can be significantly linked to parks that experience green gentrification [148]. A scoping review on green gentrification describes how the process can diminish outcomes such as psychosocial wellness, sense of community and greenway use among longtime and low-income residents from diverse backgrounds [147]. Scholars describe how green gentrification can contribute to inequities in diverse and low-income communities [149,150,151].

Green gentrification may also contribute to longtime residents from diverse communities feeling unwelcome or unsafe in new green spaces resulting in fewer visits [125,152,153,154]. For example, research in gentrifying neighborhoods along Chicago’s Bloomingdale Trail observed that perceptions of who belonged in the area led community policing efforts to target diverse youth [152]. Similarly, low-income residents may feel that new neighborhood green spaces are not for them [154] or feel dissatisfied with those green spaces [153] to the point they are not inclined to visit. Interestingly, new green spaces in gentrifying neighborhoods may have more security features which could lessen the comfortability of some residents to access them [155]. Thus, not feeling welcome and reducing visits to green spaces can reduce their potential social benefits among racially/ethnically diverse groups. Research suggests that support for new green spaces may be higher in low-income neighborhoods that are not gentrifying compared to those experiencing gentrification [154,155]. In the latter, concerns about green gentrification might lead to opposing new green amenities [154] and fewer green spaces in diverse and low-income communities [100], which can negatively impact on social cohesion.

Several studies have linked gentrification to negative mental health outcomes for longtime, low-income residents from racially/ethnically diverse backgrounds [156,157,158,159,160]. Research also shares that the mental health challenges that accompany the impacts of gentrification may strain social connections [160] and relate to poor sleep outcomes [161]. This is also important in the context of health and well-being since sleep deprivation can impair behavioral responses and social interactions [162].

In the context of gentrifying neighborhoods, green spaces may render fewer health benefits to longtime residents from racially/ethnically diverse backgrounds due to the consequences of gentrification [163]. Research in Southwest Atlanta, Georgia (USA) describes how green space redevelopment and concerns about displacement relate to sleep issues among Black adults [161,164]. However, in some cases, parks that tend to not experience green gentrification were significantly linked to social activities and cultural identity [148]. Notability, Black residents in Baltimore, Maryland had more access to parks than any other racial demographic, but this is due to “White Flight”, i.e., white households leaving diverse areas to live in majority-white suburbs [80]. Neighborhood changes may result in interactions between the ‘stayers’ and ‘movers’ that vary by attitude toward ethnic diversity [165]. To support health equity, green displacement must be addressed [74] and affordable housing cannot be an afterthought to accessorized major greening projects [151]. Green gentrification may initially desegregate places by bringing Whites and more affluent people from under-represented communities; however, the impact on social cohesion remains to be fully explored.

### 2.7. Connections among Literature Areas

**Figure 1:**
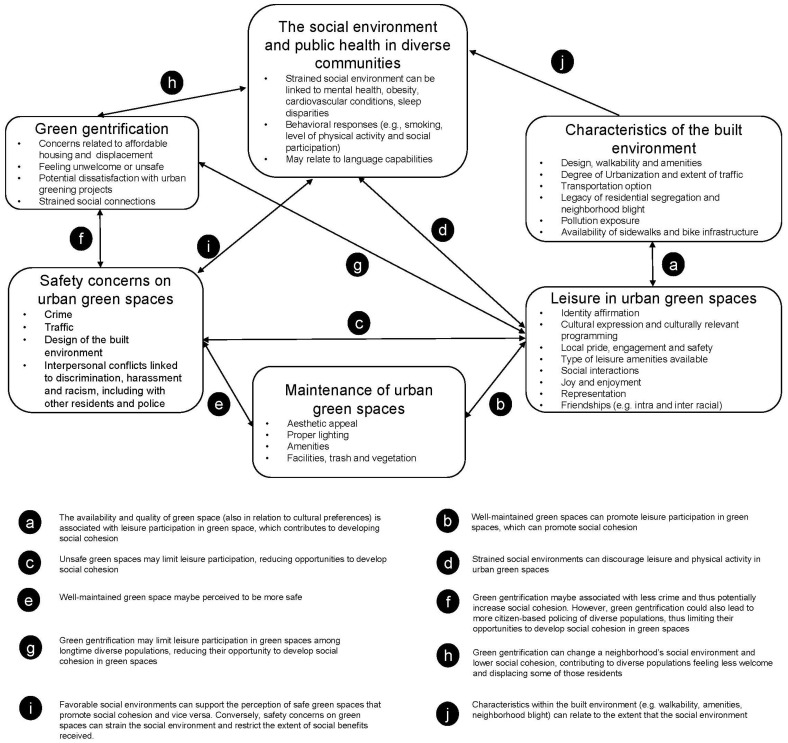
Conceptual framework to illustrate the connections between areas related to urban green spaces, social cohesion and public health in racially/ethnically diverse communities.

Based on our synthesis of these six areas, we identified several connections among the study findings, which are summarized in Figure 1. Multiple studies reinforce the importance of the social environment, as it is related to the built environment, green gentrification, leisure and safety on urban green spaces. Many arrows also connect “Leisure in urban green spaces” to other literature areas, highlighting the role of leisure activities for the promotion of social cohesion [116]. Specifically, the availability, quality, and fit to cultural preferences of urban green spaces are associated with leisure participation in such spaces (see connection a in Figure 1) [112,118], which contributes to developing social cohesion [115]. Additionally, the availability and positive characteristics of green spaces (e.g., amenities) are associated with leisure participation in such spaces (see connection b in Figure 1) [85,120], which also contributes to developing social cohesion [83]. Conversely, unsafe green spaces may limit leisure participation (connection c in Figure 1) [139], reducing opportunities to develop social cohesion [18]. Relatedly, strained social environments can limit people’s participation in leisure and physical activities in green spaces (connection d in Figure 1) [123]. Further, well-maintained green spaces can increase the perception of safety and greater visitation (connection e in Figure 1) [131].

Green gentrification is also connected to several other themes. Green gentrification may be linked with less crime [166]. However, green gentrification could also lead to more policing of diverse populations [145], thus limiting their opportunities to participate in activities that develop social cohesion in green spaces (connection f in Figure 1) [148]. Additionally, green gentrification may limit leisure participation in green spaces among longtime diverse populations (connection g in Figure 1) [147], reducing their opportunity to develop social cohesion in such spaces [160]. Also, green gentrification can change a neighborhood’s social environment and lower social cohesion, contributing to diverse populations feeling less welcome and displacing some of those residents (connection h in Figure 1) [147].

Multiple studies discussed how the social environment connects to the perception of safety which can develop [142] or hinders the social cohesion in urban green spaces in diverse communities [106,124] (connection i in Figure 1). A favorable perception of neighborhood safety and social cohesion can enhance health outcomes linked to the social environment [138]. Other connections include characteristics of the built environment and safety. For example, the built environment includes multiple features (e.g., walkability, design, amenities, neighborhood blight) that can relate to activities that develop social cohesion [69,85] (connection j). The aforementioned studies articulate how these connections are linked to multiple dimensions of public health in diverse communities.

## 3. Discussion and Future Research

In order for the health benefits of urban green spaces to be fully recognized, the social meaning of green spaces [127] and interaction with others must be taken seriously. To effectively improve social cohesion in diverse communities, disparities in the built environment, safety, leisure opportunities, green space maintenance and affordable housing should be addressed. Greater attention to the availability of quality green spaces in diverse and low-income communities is important for health and recreation policies to address health disparities [75]. Improving a green space’s social environment is also critical to support health promotion through social cohesion. As frequent experiences with discrimination are linked with less satisfaction with experiences on green spaces, this can limit the potential for urban green spaces to support health interventions [167]. Hence, exploring conditions within the social environment is critical to consider in research on social cohesion, especially in racially/ethnically diverse communities.

Given previous research on urban green spaces and social cohesion, we propose avenues for future research as it relates to racially/ethnically communities. For example, more research on the multifaceted perception of safety on green spaces [136,137] and the effect of the built environment [83] would be beneficial. Cultural settings, timing, and social context are factors that may result in variations in the way social cohesion is measured and its health implications [168]. Others argue that a ‘superficial’ or ‘quick fix’ attempt to resolve injustices by merely increasing parks in Black or low-income neighborhoods are limited [86].

Since the true inclusion of diverse perspectives is important to address underlying problems (e.g., power imbalances, redlining, and unfair policing) [86] that impact social cohesion, research to improve how this is operationalized in communities is recommended. To support inclusive socio-ecological research, it is imperative to engage disenfranchised populations in decision-making related to urban parks [86]. We propose that future research recognizes the social meaning of urban green spaces in relation to public health in diverse communities. Others observed that social cohesion can increase with age, higher education, length of residence, and presence of children in the household [69]. As scholars note that “diversity only reduces neighbor-trust among individuals who already viewed out-groups as threatening” [169], this is critical to acknowledge in future research on social cohesion and urban green spaces in diverse communities. Acknowledging such fallacies in the social environment is important to differentiate the barriers that diverse populations experience on urban green spaces.

As scholars observed differences in the access and use of parks during the early phase of the COVID-19 pandemic, exploring the post-pandemic recovery process of cities, especially in diverse communities [170], is salient. While some do not perceive trust as an indicator of formal social cohesion [171], we counter this stance and reinforce its importance to diverse communities. As social support, belonging, place attachment, and empowerment were previously identified as potential outcomes linked to social cohesion and urban green spaces [12], many studies included in this synthesis article reinforced the importance of social cohesion in diverse communities. Some argue that distrust, hostility, and a fear of competition toward people who are ‘ethnically dissimilar’ may relate to diminished social cohesion [171]. For example, to address racial inequities in environmental health sciences, Payne Sturges et al. implored researchers to consider structural racism in environmental risk assessments and to develop indicators to holistically measure racism [172]. Such insights can inform a social epidemiological approach to research on urban green spaces, social cohesion, and public health. Others also describe the importance of exploring the three dimensions (i.e., distributive, interactional and procedural) of justice in the pursuit of providing urban green spaces for social cohesion [173] and outdoor recreation [118]. As research documents potential health consequences linked to displacement concerns from green gentrification [147,150,164], interventions to support social health after urban green projects should be elevated. To support the broader vision of urban sustainability, we also recommend prioritizing an application of translational research to the nexus of urban green spaces and social cohesion, as well as to social and built environments in diverse communities. Since the maintenance of urban green spaces relates to multiple outcomes such as safety and leisure, strategies to address funding disparities of green spaces (e.g., parks) in diverse communities should be developed.

## 4. Conclusions

Urban green spaces are key factors in the interaction between the social environment, social cohesion, and health in racially/ethnically diverse communities. These experiences can be characterized by social triumph and trauma. Understanding the multifaceted ways that social cohesion is cultivated via green spaces in diverse communities can contribute to developing effective green space interventions. This can be cultivated through the authentic participatory engagement of communities, practitioners, and academic scholars who are knowledgeable about diverse communities [174,175]. Culturally informed and culturally relevant leisure experiences on urban green spaces may support positive social interactions, a sense of community and access to social support. Similar to other studies, considering race, ethnicity [176] and cultural perspectives in the planning of recreation programs and urban green spaces is important. Such efforts can support environmental justice, urban sustainability, and reduce health disparities related to social cohesion and urban green spaces. Collectively, these insights and perspectives can coalesce the interactions that all people can have within urban green spaces and their pursuit of health equity.
